# Acute Myocardial Infarction in Pregnancy: A Multidisciplinary Approach

**DOI:** 10.7759/cureus.102240

**Published:** 2026-01-25

**Authors:** Hashaam Ghafoor, Farrookh Haider, Aalami Zeba, Neeraj Kumar, Anwar Ul Huda, Amber Naz, Pramod Padmalayam, Khadija Ashraf, Osman Ahmed, Ekambaram Karunakaran, Ali O. Mohamed Bel Khair

**Affiliations:** 1 Department of Anesthesiology, ICU, and Perioperative Medicine, Hamad Medical Corporation, Doha, QAT; 2 Department of Anesthesia, College of Medicine, Qatar University, Doha, QAT; 3 Department of Cardiology, Hamad Medical Corporation, Doha, QAT; 4 Department of Cardiology, College of Medicine, Qatar University, Doha, QAT; 5 Department of Obstetrics and Gynecology, Hamad Medical Corporation, Doha, QAT

**Keywords:** acute myocardial infarction, diagnosis, maternal and neonatal outcome, multidisciplinary approach, treatment

## Abstract

Pregnancy is associated with increased hemodynamic demands on the mother’s cardiovascular system. In a normal pregnancy, most women experience symptoms such as fatigue, dyspnea, lower extremity edema, and reduced exercise ability, which can be difficult to differentiate from those of cardiac malfunction. During pregnancy, very few women experience myocardial infarction (MI). With an increasing incidence of MI during pregnancy, neonatal and maternal morbidity and mortality are significant. Particular attention is required regarding the MI cause, diagnostic assessment, and treatment plan, and post-event care is essential while treating pregnant women with MI. This review article describes the epidemiology, physiological changes, risk factors, pathogenesis, clinical presentation, differential diagnosis, diagnostic modalities, treatment strategies, time and mode of delivery, anesthesia for cesarean delivery, intraoperative anesthesia monitoring techniques, postoperative analgesia, postpartum period, maternal and neonatal outcomes, future pregnancy, case studies, and outcomes for women experiencing MI during pregnancy.

## Introduction and background

Cardiovascular disease (CVD) is the most common cause of maternal death worldwide [1]. During pregnancy, acute myocardial infarction (AMI) is linked to elevated rates of fetal and maternal morbidity as well as mortality [2,3]. However, it remains relatively uncommon. The estimated incidence, 6.2/100,000 deliveries, has been increasing during the last two decades, due to increasing maternal age and an elevated frequency of risk factors associated with CVD among pregnant women, including obesity, diabetes, and hypertensive disorders [4,5]. Furthermore, pregnancy has been identified as an independent risk factor, as pregnant women have a three to fourfold elevated risk for AMI when compared with nonpregnant females of the same age [6]. Among nonpregnant women, atherosclerosis is believed to be a major cause of AMI. However, the AMI etiology during pregnancy is varied and includes spontaneous coronary artery dissection (SCAD), coronary artery spasm (CAS), and in situ thrombus [7]. Maternal death due to AMI could be avoided with evidence-based, prompt examination of chest pain as well as AMI-optimized management [8].

## Review

Epidemiology and risk factors

Epidemiology

**Figure 1:**
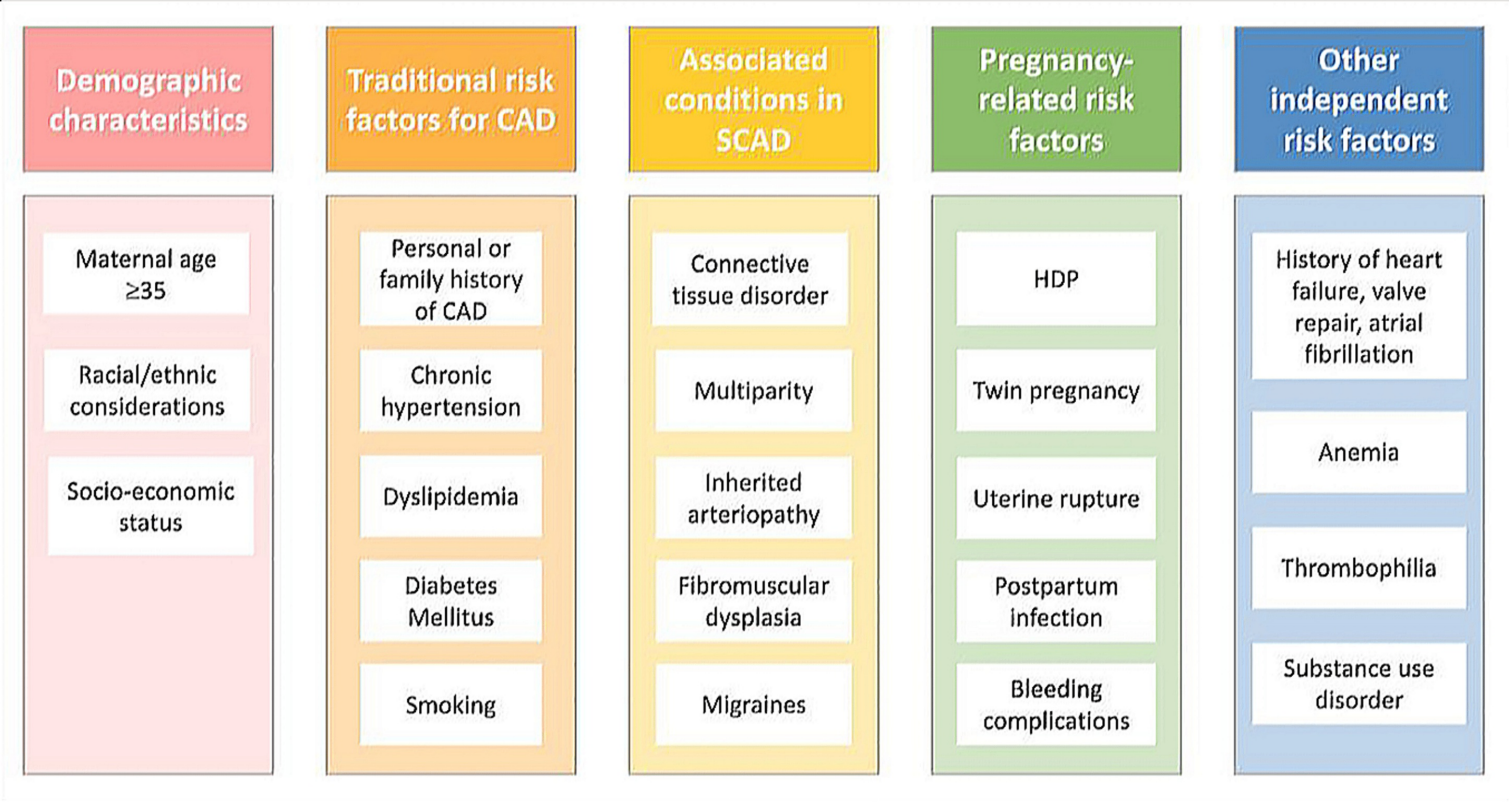
Risk factors and markers for AMI during pregnancy AMI: acute myocardial infarction Adapted/reproduced from Gédéon et al. [2], with permission

During pregnancy, the incidence of MI rises with an estimated mortality rate of 5%, which is more than that of nonpregnant females of reproductive age [9]. The risk factors for AMI during pregnancy are heterogeneous and may require special attention (Figure 1) [2].

Risk factors responsible for pregnancy-related MI are modifiable and non-modifiable. Non-modifiable risk factors likeage tend* *to increase the risk of MI as it occurs between the ages of 31 and 40 years, with an average age of 35 years, and most frequently during the antenatal and postnatal periods [10].

Other significant, non-modifiable risk factors include a history of high-risk pregnancies, relevant family medical history, multiple gestations, and ethnicity, whereas modifiable risk factors include obesity, gestational diabetes, gestational hypertension (HTN), heart failure, hyperlipidemia, multiple comorbidities, history of substance abuse, and smoking [11-13]. Among pregnant women experiencing an MI, 18.3% have experienced preeclampsia or eclampsia. The most frequent modifiable risk factors are smoking (25%) and hyperlipidemia (20%). Women with a prior history of CVD and pregnancy-related complications must be informed of their increased risk of MI during their pregnancy. MI can occur due to obstructive or non-obstructive lesions [11].

SCAD is the most common cause during pregnancy, occurring in 43% of cases. Atherosclerosis is the second most common cause, occurring in 27% of cases. A thrombus without angiographic signs of atherosclerosis was found in 17%. In 11% of cases, no cause can be found on angiography [12].

Cauldwell and coworkers reported that women with atherosclerotic disease during pregnancy are primarily older and have increased body mass index. In contrast, women with normal coronary arteries have MIs with non-obstructed coronary arteries. SCAD is the most prevalent non-obstructive cause of MI during pregnancy, with an estimated rate of 27%-43% [14].

Physiological changes during pregnancy

**Figure 2:**
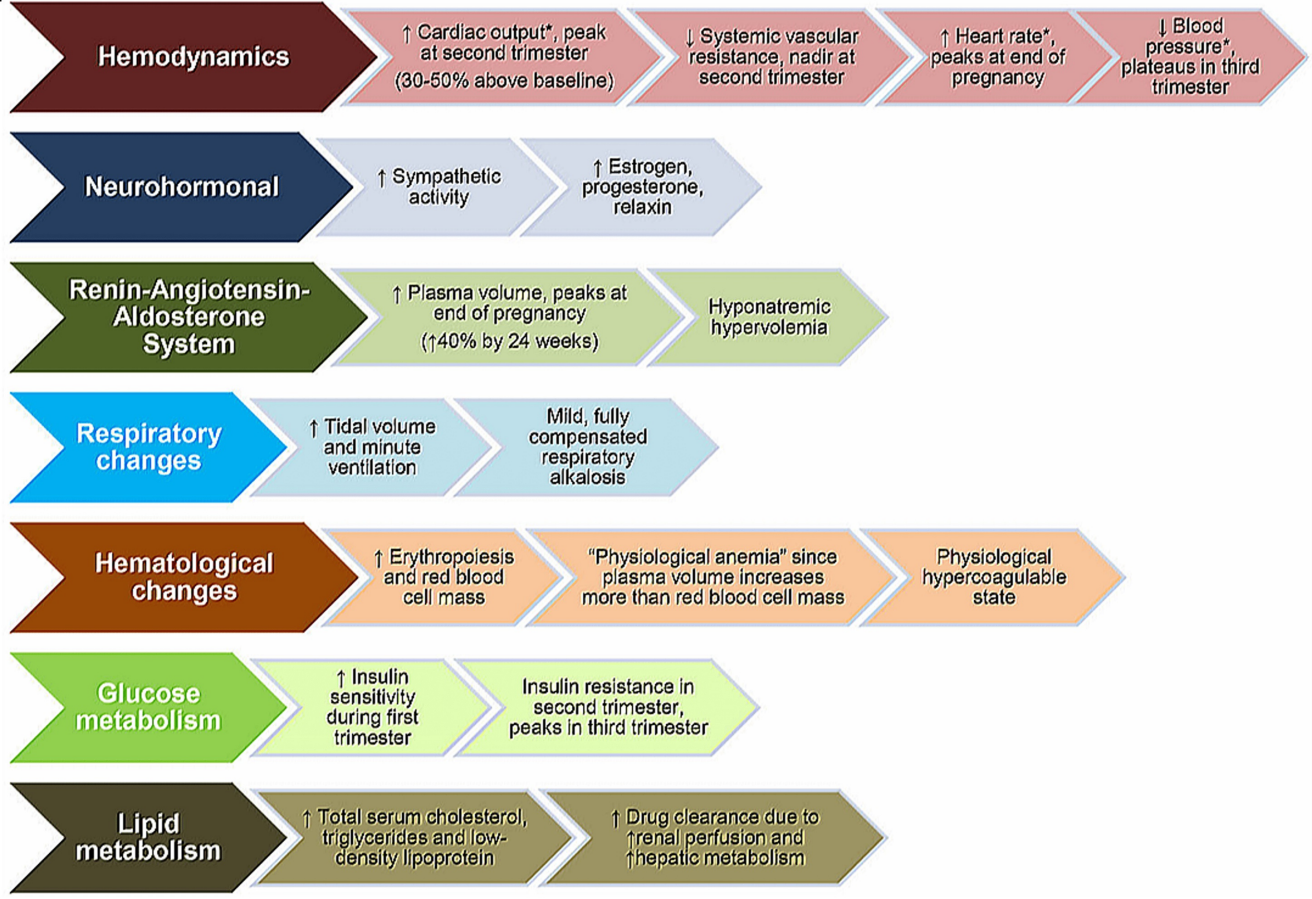
Hemodynamic, physiologic, and metabolic changes of pregnancy Adapted/reproduced from Tweet et al. [9], with permission

During pregnancy, physiological changes occur due to enhanced metabolic requirements, fetal growth, and the body’s preparation for delivery. Changes begin during the first trimester and increase by term. These changes are normally well-tolerated by healthy pregnant women, but they could be aggravated by pre-existing diseases [15,16]. Knowledge of physiological changes during pregnancy plays a significant role in recognizing the additional effects of heart disease. Pregnant women with heart conditions respond differently to the normal physiological alterations of pregnancy depending upon their specific disease as well as its severity [17]. Multiple systems in the body are affected by the physiological alterations of pregnancy [15]. These hemodynamic, physiologic, and metabolic changes of pregnancy are illustrated in Figure 2 [9].

Cardiovascular System

Changes in the cardiovascular system during pregnancy are important and occur as early as eight weeks of gestation. They include a compensatory fall in systemic vascular resistance (SVR) of 25%-30% with enhanced cardiac output (CO) of 40% [18]. Such changes, and other factors, could affect individuals with aortic stenosis and right-to-left shunting [19]. Arterial pressure and mean arterial pressure decrease transiently regardless of the rise in CO, and the renin-angiotensin system is accentuated, causing a rise in the plasma volume from sodium and water retention [20]. Increases in the intravascular volume of up to 50% are observed during the third trimester, and individuals with fixed CO states, such as those with valvular heart disease and myocardial dysfunction, tolerate it poorly [19].

Respiratory System

The consumption of oxygen is enhanced by 20%, and the metabolic rate is increased by 15% during pregnancy, which leads to an enhanced demand for oxygen. Due to the increased tidal volume, ventilation is enhanced by 40%-50%, and it results in hyperventilation, respiratory alkalosis, decreased carbon dioxide partial pressure, and increased arterial oxygen partial pressure [18]. During the later stages of pregnancy, the uterus is displaced upward while esophageal sphincter tone is diminished. Both of these increase intragastric pressure and the risk of aspiration [20].

Hematological System

Physiological anemia during pregnancy is due to an increased plasma volume (40%-50%) with only a 20% increase in erythrocyte volume, which facilitates uteroplacental blood supply [16]. During pregnancy, the platelet count normally decreases due to increased platelet destruction and hemodilution, although it remains in the range of 100-150 × 10^9^ cells/L [18,20]. During pregnancy, hypercoagulability exists to prevent excessive blood loss during placental detachment. Anticoagulation could be indicated, particularly among individuals at increased risk for thrombosis [19].

Central and Peripheral Nervous System

Generally, the epidural plexus is engorged due to increased venous pressure beneath the pregnant uterus. This leads to a reduction in the epidural space as well as a compensatory cerebrospinal fluid drop that causes a 25% decrease in the requirement for local anesthetic as well as an extended time of its action [20].

Renal and Hepatic System

During the period of pregnancy, renal blood supply is enhanced by 50%, and the glomerular filtration rate is increased from 100 to 150 ml/minute. This results in reduced blood urea nitrogen (BUN) and serum creatinine levels [16,20]. During pregnancy, changes in liver function are temporary, but pregnancy-related liver disorders such as eclampsia, hemolysis, and elevated liver enzymes could be lethal [21].

Pathogenesis

An AMI associated with pregnancy could have multiple etiologies [11]. SCAD plays an important part and is responsible for up to 43% of cases [22]. Currently, coronary artery disease (CAD) is believed to be the second most significant cause of MI in pregnant women [14,23]. Coronary spasm and thrombosis without CAD are very uncommon etiologies of an MI [22].

SCAD

SCAD primarily affects young, white women (83%), with an insignificant change in baseline characteristics between antenatal and postnatal women suffering from SCAD having been found [24]. Similar to coronary spasm, the SCAD primarily takes place during the postpartum period among multiparous women. In the antepartum period, it primarily presents during the third trimester [25]. The SCAD timing could be associated with cardiac stress due to swift uterine contraction after the delivery, as well as the return of significant blood volume to systemic circulation [26]. Physiological modifications related to gestation and labor hemodynamic effects can precipitate SCAD as well, mostly when the predisposing conditions, for example, fibromuscular dysplasia, coexist [27]. Twin pregnancy and pre-existing and gestational HTN could enhance shear stress and endothelial dysfunction, thus stimulating the onset of the SCAD and severe aortic dissection [28,29]. However, the chronic disease incidence as risk factors, for instance, chronic renal disease and diabetes mellitus (DM), is notably found to be lower among pregnant women with SCAD, with <61% of such women not showing any evidence of SCAD [22,25]. An excess of progesterone appears to cause the breakdown of elastic fibers as well as a reduction in the acid mucopolysaccharide ground substance, and estrogen could enhance matrix metalloproteinase release [11]. Moreover, a significant proportion of non-pregnant women affected with SCAD use oral contraceptive pills, which validates this observation [30,31].

CAD

Pregnancy-associated AMI due to CAD occurs at a similar frequency during both the antenatal and postnatal periods [22]. In an investigation of the European registry, it was found that pregnant women with CVDs reported that CAD occurred more frequently than SCAD. Coronary thrombosis without CAD results in a comparatively high percentage of AMIs associated with pregnancy; however, in those not pregnant, it is much more uncommon [32,33].

Coronary Spasm

Preeclampsia is a significant risk determinant for coronary spasm, as it leads to systemic endothelial impairment due to an imbalance in thromboxane and endothelin secretion [34]. During pregnancy, several other mechanisms, including increased vascular reactivity to noradrenaline and angiotensin II, as well as the release of angiotensin and renin due to reduced uterine perfusion in the supine position, are also risk factors for coronary spasm [35].

It can be quite challenging to recognize an AMI phenotype among young women. One study reporting on the mechanism of AMI noted that, based on the recent universal definition of MI, 10% of patients remained undiagnosed [36].

Clinical presentation

Most MI patients during pregnancy (75%) experience ST-segment elevation MI (STEMI), while the remaining proportion (25%) experience non-STEMI [12]. The majority of MIs occur during the third trimester (25% STEMI and 32% non-STEMI) or during the postnatal period (45% STEMI and 55% non-STEMI) [22]. Primarily, the anterior wall is involved in 69%-78% of patients, the inferior wall in 27%, and the lateral wall in 4% [10]. An increased percentage of anterior wall involvement has been observed in several reports describing MI among pregnant women, regardless of the primary reason for MI in each study (atherosclerosis vs. SCAD) [12,37]. This is a significant finding that explains the comparatively increased frequency of catastrophic presentation, as well as MI complications, during pregnancy. Left ventricular ejection fraction (LVEF) waslower than 40% in 54% of patients, 30% in 24% of patients, andlower than 20% in 9% of patients. Cardiogenic shock or heart failure occurred among 38% of females and ventricular arrhythmias among 12%, while AMI/recurrent angina occurred among 20% of females [22].

In a major case review of Elkayam and colleagues, SCAD-associated MI during gestation involved left main coronary artery (LMCA) among 26% of patients, the left anterior descending (LAD) artery among 34%, the left circumflex (LCX) artery among 3%, and right coronary artery (RCA) among 14%, while multiple vessels among 39% of patients [22].In comparison, the vascular distribution observed among non-pregnant women most commonly involved vessels were the LAD (61%), followed by the RCA (25%), LCX (25%), and the LMCA (4%) [38]. Similar findings were observed regarding SCAD in both non-pregnant and pregnant women. The findings of a study involving pregnant women reported that 81% were affected by STEMI, while 25% were hemodynamically unstable on presentation. Among pregnant women, 26% needed intra-aortic balloon pump support, while 14% needed advanced cardiac life support [25]. It is comparable with the presentation among non-pregnant women, in which 80% to 90% of women present with acute coronary syndrome (ACS) and nearly 40% with STEMI, while 40% present with non-STEMI. Almost 10% to 13% of episodes are complex due to a heart attack, while cardiogenic shock takes place among 2% to 4% of patients [38,39].

During the first trimester, the causes of MI are usually atherosclerotic MI, especially if the patient has vascular risks. During the second trimester, the most common causes of MI are thrombosis and atherosclerotic MI. During the third trimester, SCAD is considered the most common cause for MI associated with pregnancy, while an insignificant proportion of ACS is due to coronary spasm in this period. SCAD is the most common cause of AMI in pregnancy, representing up to 27%-43% of cases [14]. Atherosclerotic CAD can appear in any trimester, and it is more common during the antenatal period, while SCAD primarily takes place near or soon after delivery [12,40].

Several hemodynamic modifications that take place during pregnancy can be leading factors for SCAD in addition to individual predisposition. These modifications include a rise in CO and plasma volume by 50% and 40%, respectively. Increased heart rate is an important ischemic factor and can considerably increase the requirement for myocardial oxygen. During the period of labor and childbirth, numerous factors increase cardiac oxygen requirement. CO increases by 15% during premature labor, 25% in stage 1, and a 50% increase in stage 2. The maximum rise in CO is observed during stage 3 of labor. This is possibly why the majority of pregnancy-related SCADS are observed during the early postpartum period [12,23].

Differential diagnosis

**Figure 3:**
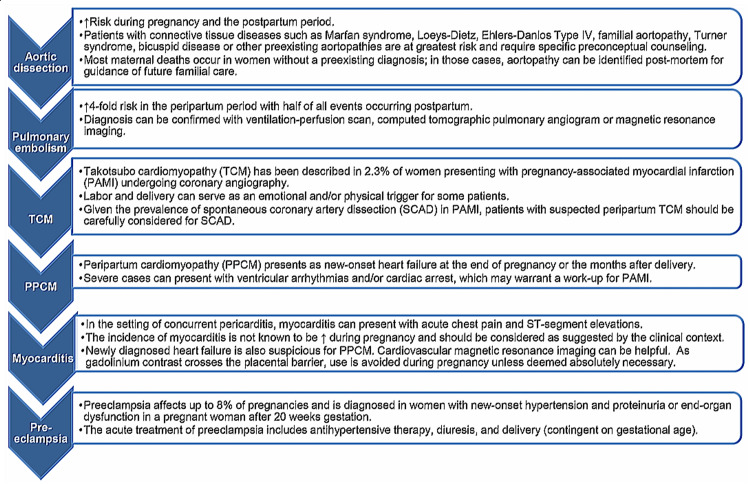
Differential diagnosis of pregnancy-associated myocardial infarction TCM: Takotsubo cardiomyopathy; PPCM: peripartum cardiomyopathy Adapted/reproduced from Tweet et al. [9], with permission

Among pregnant women who have chest pain, the differential diagnosis primarily comprises preeclampsia, pulmonary embolism (PE), and aortic dissection [11]. All of these diagnoses have an increased prevalence during the third trimester and the early postnatal period, where the majority of cardiac events also occur. These events could result in a rise in the cardiac troponin levels that may make identification even more difficult [41]. In particular, pregnancy enhances the PE risk almost fourfold, obligating physicians to carefully exclude it [42]. Given its epidemiological significance, the venous thromboembolism risk evaluation is suggested for all females either before pregnancy or during the first few weeks of pregnancy [41]. Several grave conditions could present with the same clinical features as pregnancy-associated MI (PAMI), sometimes accompanied by an abnormal ECG and elevated biomarkers (Figure 3) [9].

The common clinical characteristics of PE, dyspnea, pleuritic chest pain, hemoptysis, and palpitation are likely to be associated with limb pain due to deep vein thrombosis. However, most of these signs and symptoms could also be associated with physiological gestation, which makes the diagnosis difficult [43]. An ECG may demonstrate tachyarrhythmias and sinus tachycardia, the S1Q3T3 sign, as well as right ventricular strain, while a transthoracic echocardiogram could identify right ventricular dilation and increased pulmonary arterial pressure. However, the precision of these investigations is lower among pregnant women. The utilization of D-dimer plasma levels and Wells’ score, which is generally important for the estimation of PE probability, has not been confirmed during pregnancy. Hence, currently, the best diagnostic technique for these women remains uncertain [44]. At times, a venous ultrasound may show deep vein thrombosis, while an MRI could adequately identify pulmonary circulation. However, a low-dosage computed tomography angiography should be carried out if the clinical doubt remains when the abovementioned studies have not verified the diagnosis [41].

Diagnostic modalities

Electrocardiogram (ECG)

There are normal variations that occur in the ECG during pregnancy. They are due to physiological alterations during pregnancy, particularly spatial positioning of the heart and myocardium, and electrical alterations due to hormonal as well as sympathetic alterations [2]. The ECG normal variations found during the period of pregnancy comprise an insignificant deviation of the left-axis, minute, non-pathological Q-waves within anterolateral and inferior leads, T-wave inversions within anterior leads and lead III [45], as well as sinus tachycardia and ventricular and supraventricular ectopic beats [46,47]. Therefore, small Q-wave and mild, non-specific ST-segment and T-wave modification should be carefully interpreted in the ECG of pregnant women. The presence of an elevated ST segment, T-wave, and left bundle branch block requires rapid examination as well as a strong level of ischemia suspicion [48].

Circulating Biomarkers

High-sensitivity cardiac troponin I and troponin T are useful biomarkers to identify cardiomyocyte damage among non-pregnant women [49]. Unlike creatine kinase and creatine kinase-MB isoenzyme, which are released during labor through the placenta and uterus, the cardiac-related troponins remain unaffected by normal pregnancy and labor, making them reliable biochemical markers for identifying myocardial ischemia among pregnant women [50]. A study carried out among 880 pregnant women demonstrated that the troponin-I levels were found to be higher among those with preeclampsia and gestational HTN than among normotensive controls, highlighting the stress placed on the heart due to these conditions [51]. Also, the troponin-T levels obtained on four occasions during and after pregnancy were found to be relatively higher during the peripartum period and remained less than the cutoff levels for excluding myocardial ischemia [52]. Hence, it seems that cardiac troponin levels indicate a slight rise in pregnancy-associated HTN [51-53], and they remain helpful biological markers for the diagnosis of AMI during gestation. The existing cutoff values for non-pregnant women are suitable to exclude cardiac ischemia during pregnancy [52].

Imaging Modalities

Transthoracic echocardiography (TTE) is the most frequently utilized, non-surgical modality for cardiac imaging during pregnancy, due to its accessibility, safety, and less radiation exposure [41]. Alterations in cardiovascular physiology associated with pregnancy could cause ventricular remodelling due to a rise in the right and left ventricular mass, end-diastolic ventricular volume, and enlargement of the left atrium, while increasing the wall thickness [54]. Also, pregnant women with HTN during pregnancy develop enhanced left ventricular mass as well as relative wall thickness [55]. In spite of adaptive cardiac remodelling, identification of abnormalities in wall motion can confirm ischemia and requires further examination [54,56]. Although the TTE role in evaluating a possible STEMI is usually carried out following percutaneous coronary intervention (PCI), delay should be avoided during transfer to the catheter laboratory [2].

The computerized tomography coronary angiography (CTCA) is a non-surgical imaging method that aids in coronary artery visualization [57]. It is readily accessible and avoids the risks of procedure associated with coronary angiography (CA), for example, an extension of SCAD. CTCA has demonstrated variable success for the identification of SCAD, and satisfactory beta-blockade is required for better imaging as well as identification of coronary dissection [58]. Although there are issues concerning potential fetal radiation exposure, it has been confirmed that the levels remain less than the accepted cumulative dosage [59]. CTCA is an important investigative tool in patients when the SCAD is strongly suspected, where the intervention need is unclear, or if there is doubt about the coronary etiology for the patient’s symptoms [60].

Diagnostic CA

CA is a definitive technique for diagnosing and managing ACS [61]. Minimizing deep catheter insertion, restricting guidewire movement, and reducing the frequency of injections with low-pressure contrast can help in lowering the coronary dissection risk [62]. The radial access is favored to minimize bleeding, and the females should be placed in the left lateral decubitus position or supported with a wedge to avoid aortocaval compression [48].

Reluctance to pursue this intervention could arise from concerns regarding potential fetal risks associated with ionizing radiation. Investigating CA reveals the fetus to approximately 1.5 mGy, while the PCI increases the dosage to approximately 3 mGy, both of which are well below the accepted threshold for severe fetal radiation exposure [2]. Strategies to reduce the radiation exposure comprise prioritizing upper limb access, applying abdominal shielding, reducing frame rate to 7.5 frames/second, utilizing low-dosage fluoroscopy focused on the field of concern, limiting fluoroscopy duration, adjusting the radiation beam, utilizing wedge filters, choosing anteroposterior forecasts, and positioning the intensifier as close to the female as possible [30,41,63-66]. While abdominal shielding reduces fetal radiation exposure, it can also increase scattered radiation and should be implemented in a manner that does not compromise the effectiveness of the intervention [41]. According to the European guidelines (2018) regarding CVD during pregnancy, CA and PCI should be carried out on pregnant women when the standard symptoms are present [41,67].

Treatment strategies

PCI

PCI is advisable for individuals with ST elevation or elevated risk [10]. It is essential to know the radiation exposure among pregnant women to minimize radiation risk. If possible, the procedure should be delayed until the completion of major organogenesis, between two and seven weeks of gestation. When CA is necessary, the radiation dose must be kept as low as possible, ideally <50 mGy. Diagnostic CA exposes the fetus to about 1.5 mGy, and PCI raises the dose to 3 mGy, which is still within the recommended range for fetal radiation exposure. The effects of doses between 50 and 100 mGy on the fetus are considered to be uncertain. Doses between 100 and 200 mGy can cause radiation-induced abnormalities, including growth retardation, intellectual abnormalities, malignancies, and neurological effects. It is recommended to terminate the pregnancy if the fetal radiation dose is >150 mG [2,11,12].

Treatment varies depending on the AMI etiology. Individuals suffering from atherosclerotic disease are found to be at lower risk compared with those with SCAD as their underlying etiology, due to higher complication rates, and almost one-third of PCI procedures are successful in SCAD patients [68,69]. Catheter-induced dissection can occur due to arterial wall fragility, causing additional ischemic injury as well as severe heart failure, which may require a coronary artery bypass graft and mechanical circulatory support [65,69]. Hence, PCI should be avoided in patients with stable hemodynamics; instead, appropriate treatment is essential. However, for patients with an elevated risk of MI, ST-segment elevation, and constant chest pain, emergency invasive strategies must be employed [9,65].

Thrombolysis

Thrombolysis is not advised as a revascularization strategy and should be considered only as a last resort, due to an elevated risk of bleeding, including fetal and maternal hemorrhage [69]. Among individuals with SCAD as their underlying etiology, the risk for dissection flap proliferation and intramural hematoma expansion should be a cautionary warning [68]. Significant bleeding has been observed in 18%-58% of cases during pregnancy and the postpartum period [70,71]. One study, which included 28 pregnant women receiving fibrinolytic therapy for multiple indications (PAMI, PE, and stroke), identified two deaths (7%) and three unfavorable events (11%), including abdominal and brain hemorrhage [10].

Drug Therapy

During pregnancy, MI treatment is necessary as it will improve maternal outcomes and fetal survival. Full-dose aspirin can be utilized at less than 32 weeks of gestation, while 81 mg can be utilized throughout the entire pregnancy [9]. Clopidogrel is the P2Y12 preferred inhibitor, but it must be discontinued at least seven days before neuraxial anesthesia [68,72]. However, both prasugrel and clopidogrel can be used during pregnancy. The use of clopidogrel is unclear during pregnancy, as it may cause an increased risk of dissection extension if SCAD is the primary etiology, a risk of hemorrhage, and potential evidence of insignificant benefit [72]. Ticagrelor is not advised during lactation, as an increased risk of bleeding has been noted among animals. Dual antiplatelet therapy (DAPT) could be administered to patients waiting for intracoronary stenting, while for individuals with SCAD, single-agent aspirin is preferred [12,73]. It is considered safe to use all the anticoagulants during pregnancy and breastfeeding if really needed. Among the glycoprotein receptor blockers, abciximab is safer to use compared to eptifibatide and tirofiban, especially when breastfeeding (Table 1) [12].

**Table 1:** Safety profile of cardiac medications during pregnancy and lactation Adapted/reproduced from Edupuganti et al. [12], with permission

Drug	Pregnancy (human vs animal studies)	Lactation (human vs animal studies)	Recommendations
Thrombolytics			
Alteplase (10 case reports)	Its high molecular high weight likely prevents transfer to the embryo	It is unknown whether the drug crosses the placenta or is excreted into breast milk	Compatible with pregnancy & lactation
Reteplase (one case report)		Given the clinical indication and short half-life, use during lactation is unlikely to cause significant adverse effects. Plasminogen activators are unlikely to cross into breast milk	
Urokinase (six case reports)	It is unclear whether it crosses the placenta. Placental proteinase inhibitors may inactivate reteplase	Given its indication and short half-life, use during lactation is unlikely to pose a significant risk	Low risk in pregnancy and probably compatible in lactation
Tenecteplase (no human data available)			
Antiplatelet medications			
Prasugrel	There is no human data available	No human data	No human data is available. The animal data suggest low risk. It may be used during pregnancy if the benefits outweigh the risks. Possibly compatible with lactation
Ticagrelor	There is no human data, but animal data suggest a risk to the fetus	No human data available	No human data is available, while animal data suggests the risk. If considered in pregnant patients, risks should be evaluated on a case-by-case basis. Breastfeeding may be associated with potential toxicity
Clopidogrel	Limited data is available on humans, and not teratogenic in animals	Human data is unavailable on breastfeeding	Limited data is available on humans. Probably, it is compatible with pregnancy and lactation
Aspirin	With full-dose aspirin, human data suggest increased risk during the first & third trimesters. It could be linked to IUGR, teratogenic effects, and hemorrhage. It can delay labor, and premature closure of the ductus arteriosus may take place among premature infants	Limited data is available on humans. Potential for salicylate poisoning	Low-dose aspirin is generally considered compatible with pregnancy. Limited human data propose potential toxicity among breastfeeding infants
Anticoagulants			
Heparin	Does not cross the placenta	Does not excrete in the breastmilk	Compatible with pregnancy & lactation
Bivalirudin	Human data is not available, while animal data shows a low risk	Human data is not available	It could be utilized during pregnancy if the benefits compensate for the risks. Possibly compatible with lactation
Fondaparinux	Limited data is available on humans	Human data is not available	It can be utilized if advised during pregnancy. Possibly compatible with lactation
Enoxaparin	Safe during pregnancy	Human data is not available	Compatible with pregnancy & lactation
Glycoprotein receptor blockers			
Abciximab	Does not reach the fetus in clinically substantial amounts	Improbable to be released in breastmilk	Compatible with pregnancy and lactation
Eptifibatide, tirofiban	Limited data is available on human while animal data shows low risk or reassuring	The drug is likely degraded in the infant gastrointestinal tract; however, clinical data are limited	Limited data is available on humans. Animal data shows low risk or being compatible with gestation. Preferably to withhold breastfeeding

During pregnancy, the use of heparin is considered safe due to its rapid onset, brief half-life, and the ability to adjust the dose easily by measuring the clotting activation time [64]. Heparin has no teratogenic effects and cannot cross the placenta; however, it can cause thrombocytopenia in 3% of patients and should not be used 4-6 hours before childbirth [12,69]. Heparin with a low-molecular weight has benefits over standard heparin through better bioavailability and reduced affinity for heparin-binding proteins, and cessation of low-molecular-weight heparin 24 hours before childbirth is required. If necessary, treatment could be resumed after delivery. Neither low-molecular-weight heparin nor heparin is secreted in breast milk, making them both safe for breastfeeding [69].

PCI, along with revascularization, is considered the gold standard for treating ACS. Interventional cardiologists and obstetricians concerned about the radiation risk for developing a fetus may hesitate to recommend PCI [64,74]. Currently published evidence does not fully define the magnitude of radiation risk during gestation. The decision for PCI can be influenced by the concerns of both the pregnant woman and her healthcare professionals regarding radiation-related procedures. During pregnancy, PCI can safely be carried out, and it is a relatively safe procedure that should be undertaken once it is indicated [35].

If PCI is carried out, the choice of stent, whether to utilize a bare metal stent (BMS) or a drug-eluting stent (DES), is crucial. Special attention is necessary to reduce the time duration for DAPT to decrease the risk of bleeding [68]. The BMS is advised for women in their third trimester, as a shorter duration of DAPT, just four weeks, is required [12], while a DES requires DAPT for at least three months following implantation. During the first two trimesters, the DES is advised [9]. DES is preferred for non-pregnant, adult women as it decreases the risks of MI as well as the need for repeat revascularization compared with the BMS [69].

Guidelines for standard revascularization must be followed when performing PCI in the presence of atherosclerotic lesions [41]. If a coronary stent is indicated, DES should be utilized to ensure a low rate of restenosis and permanent coronary patency [41], while drug-eluting balloons could be considered as an alternative therapy [75]. This is particularly significant for pregnant women who are younger than the general revascularization patients. Newer model stents facilitate a shorter duration of DAPT following DES implantation, with ischemic event rates comparable to those of BMS. The timing of delivery is crucial following stent implantation, as DAPT increases the risk for bleeding, particularly when combined with neuraxial anesthesia, as well as the potential for epidural hematoma [72].

Patients should receive care in an ICU equipped for thorough maternal and fetal monitoring as well as obstetric care, along with management guided by a multidisciplinary team comprising obstetricians, cardiologists, neonatologists, and anesthesiologists. The best time for performing PCI therapy is during the second trimester, after the fourth month, as per European Society of Cardiology guidelines [72]. This is due to the completion of fetal organogenesis, fetal thyroid inactivity, and the small size of the uterus at this stage of pregnancy, which allows for a larger distance between the fetus and the chest compared to later during pregnancy.

Gestational age, diagnostic techniques, estimated fetal dosage, and most significantly, the understanding that fetal life depends on the mother's life, should all be considered [41,75]. It has been found that PCI benefits outweigh the minimal risk of radiation exposure to the fetus and mother, as long as necessary precautions are taken to minimize the radiation doses for both. Moreover, a patient-specific risk-benefit analysis of drugs utilized in the cardiac catheterization lab (such as aspirin, nitroglycerin, anticoagulants, clopidogrel, calcium channel, and beta-blockers) should be performed instead of using previously defined categories of pregnancy risk [76].

Beta-blockers aid in reducing both the consumption of myocardial oxygen and shear stress during SCAD, and they have also been utilized during pregnancy to manage HTN, Marfan syndrome, myocardial ischemia, and mitral stenosis [11,77]. During pregnancy, propranolol is safe. Metoprolol is much more effective when utilized for ventricular arrhythmias and supraventricular tachycardia during pregnancy, while labetalol is utilized as a first-line treatment for both acute and chronic HTN during pregnancy; however, dosage adjustment is required due to a shorter half-life. Neonates exposed to beta-blockers exhibited increased rates of hypoglycemia and bradycardia, and their use has been linked to fetal growth retardation [72,78,79].

Nifedipine belongs to the class of calcium channel blockers that are primarily utilized during the treatment of HTN, eclampsia, and tocolysis. Verapamil is a second-line medicine for rate control in atrial fibrillation, as well as for treating idiopathic sustained ventricular tachycardia among pregnant women [69,79,80]. These medicines are emitted in human milk and are not recommended for breastfeeding women [69]. Angiotensin receptor and angiotensin-converting enzyme blockers are contraindicated due to their fetotoxic effects [11]. Nitrates can help in alleviating chest discomfort or treat coronary vasospasm, but caution is needed due to maternal hypotension as well as placental hypoperfusion. Nitroglycerin is suggested when preeclampsia is linked to pulmonary edema [68,81].

Statins should not be used for 1-2 months before pregnancy, as well as in early pregnancy, due to their teratogenic effects. Although a recent study reported no clear association between statin use during pregnancy and congenital abnormalities, due to the limited data available, it is still advisable to discontinue statins before conception [75,82].

Timing of delivery

For females with AMI during pregnancy, selecting the accurate timing for delivery plays an important part [83]. Delivery timing depends on gestational age and maternal stability. Delivery should be delayed for at least two weeks after the infarction, as it will decrease the hemodynamic stress following the event [41]. A premature birth has been linked to an enhanced risk of reinfarction and significant mortality [2]. Better cardiac function was observed following two weeks of medical therapy after MI, and pregnancy is usually continued until 34 weeks of gestation [83]. If the fetus is viable, premature delivery may be induced in recurrent SCAD cases. Induction using drugs like dinoprostone and misoprostol should be avoided if vaginal delivery is desired, since they can increase the risk of coronary spasm, especially in SCAD. Beta-blockers that do not interfere with the uterine contractions could be continued without risk [12].

Mode of delivery: vaginal or cesarean delivery

There is no consensus regarding the preferred delivery mode for pregnant females following an acute MI [83]. A vaginal delivery has the advantages of less bleeding and a low risk of infectivity, thromboembolism, and venous thrombosis and should be suggested for the majority of women [41]. Mihaljevic et al. presented two cases of women with PAMI, one of whom underwent vaginal birth, given her stable clinical status [84]. A C-section seems to be the most manageable delivery technique for the parturient experiencing a severe cardiac event, as it provides good control throughout and helps in preventing stress responses due to a protracted vaginal delivery. During pregnancy-onset ischemic heart disease (IHD), the frequency of C-sections is 62%-84.6% [31,85]. Generally, elective C-sections do not enhance fetal or maternal outcomes among females who have stable cardiac disease. In a study, Baris et al. reported that elective C-sections should be performed primarily for high-risk obstetric indications, for example, following a recent acute MI or if LVEF continues to decrease [31]. The unfavorable cardiovascular events risk was notably higher in the C-section group compared with the vaginal delivery cohort [86], highlighting the importance of a multidisciplinary team focusing on maternal recovery following surgery. In a review, Roth et al. reported that the delivery mode among women with PAMI must be evaluated by obstetrical signs as well as the mother’s clinical status [87]. An individualized and appropriate decision based on the patient’s clinical condition is essential for determining the optimal delivery mode.

Anesthesia for vaginal delivery

For the majority of women with CVD, vaginal birth, along with efficient neuraxial analgesia, is considered the best delivery method [88-90]. Vaginal delivery, as compared with C-section, is linked to insignificant bleeding, rare wound infections, and rare thromboembolic events [91-93]. Neuraxial analgesia reduces maternal plasma norepinephrine and epinephrine levels in labor [94]. Hence, the placement of an epidural catheter at the onset of labor pain is the usual approach. Also, the epidural catheter serves as a conduit for transitioning to surgical anesthesia if an emergency C-section becomes necessary [95]. It is advisable to quickly replace the epidural catheter if it ceases to provide adequate labor analgesia [96].

For labor pain control, neuraxial catheters can be placed using the epidural and combined spinal-epidural (CSE) techniques. Utilizing saline instead of air during the loss-of-resistance method for epidural catheter placement may reduce the venous air embolism risk if the needle inadvertently enters a blood vessel. This is particularly significant for patients who have an intracardiac shunt, among whom the paradoxical embolism risk exists [97,98]. Among females with CVD, the risks as well as advantages of conservative epidural test dosage should be carefully evaluated [96]. For individuals who are at risk of decompensation due to the quick onset of inadvertent intravascular epinephrine or spinal anesthesia, the epidural test dosage may be divided, followed by motor, as well as sensory, evaluation to assess its effects. When utilizing a CSE method for labor analgesia, intrathecal medications at lower dosages, for instance, 2 mg of bupivacaine together with 10 µg of fentanyl, are successful. A slow aspiration using a low-volume syringe (1-3 mL) could help in identifying the intravascular catheter or inadvertent intrathecal placement. Among cardiac individuals, the initial administration of epidural medication for labor analgesia should be performed gradually over 10-20 minutes, with vital signs continuous monitoring and careful assessment of both motor and sensory blockade. This allows for the identification of dislocated catheters and offers time to manage and prevent hypotension [88]. Labor analgesia could be maintained with a local anesthesia, bupivacaine 0.0625%-0.125% with 1-2 µg/mL fentanyl, administered through either patient-controlled epidural infusion or planned intermittent boluses. Both methods may also include patient-controlled epidural dosages [99-101].

Irrespective of the method utilized for the initiation of neuraxial anesthesia, it is important to maintain baseline hemodynamic afterload to ensure coronary perfusion, especially among patients with left ventricular hypertrophy and pulmonary HTN [88]. Monitoring of intra-arterial pressure could be beneficial for tracking maternal blood pressure (BP) and allowing for prompt treatment of any deviation from baseline [102]. For individuals with CVD who are found to be at an elevated risk of pulmonary edema, anesthesiologists should avoid the routine use of prophylactic fluid bolus before starting neuraxial labor analgesia. Once neuraxial analgesia begins, hypotension must be managed with vasopressor support to maintain the BP at baseline levels [103]. An insignificant volume of crystalloid supplementation (200 mL) could be utilized to manage mild hypotension; however, vasopressor support must be the primary approach to increase SVR and to maintain maternal BP if it drops due to neuraxial anesthesia. It is common practice for patients to receive intravenous phenylephrine (50-100 µg) to maintain SVR. A constant infusion of phenylephrine may be utilized as needed among laboring women to maintain their BP at baseline levels [88]. Other vasopressors, for example, vasopressin or norepinephrine, and inotropic medicines, namely, dobutamine, epinephrine, milrinone, and dopamine, could be given less frequently during vaginal childbirth [104]. Among patients who suffer from an intracardiac shunt, where the low afterload could cause hypoxemia, it is essential for anesthesiologists to promptly address afterload reduction using vasopressor support [88].

Anesthesia for cesarean delivery

Generally, neuraxial anesthesia is recommended for C-sections, including among females with maternal World Health Organization (mWHO) class III/IV lesions. However, the choice of anesthetic method must be tailored to the individual patient [105,106]. Possible reasons for opting for general anesthesia (GA) include cardiopulmonary decompensation requiring intubation, contraindications to neuraxial anesthesia, for example, active anticoagulation, acute thrombocytopenia, and maternal rejection of neuraxial methods [107,108]. For patients vulnerable to decompensated heart failure, a potential risk is found for hemodynamic instability directly after the delivery of the baby due to abrupt autotransfusion from aortocaval decompression, as well as uterine contraction following delivery. If a pregnant patient with CVD experiences hypoxemia or dyspnea and is not able to lie supine before C-section, GA with intubation could be necessary to manage the cardiopulmonary decompensation risk immediately following delivery. Among these patients, arterial monitoring is employed to rapidly detect and address fluctuating hemodynamic changes due to volume shifts. The hemodynamic effects of spinal anesthesia for C-section are faster and more pronounced compared with those of an epidural anesthetic administered gradually over 15-20 minutes [109]. Individuals with mWHO class I/II cardiac disease generally tolerate a standard intrathecal dosage of local anesthetic, such as 12.5 mg of hyperbaric bupivacaine, for C-section delivery. For pregnant patients with mWHO class III/IV cardiac lesions, more gradual onset sympathectomy could be beneficial. Depending upon the specific cardiovascular condition, the alternatives include epidural anesthesia, a CSE approach with intrathecal opioids, or the sequential CSE method. In the sequential CSE method, intrathecal opioids and bupivacaine at a low dose (2.5-5 mg) are administered first; then, gradual titration of epidural local anesthetic, normally 2% lidocaine, is administered to attain surgical level T4-T6 [110]. The sequential CSE method theoretically offers the advantage of combining the more reliable, symmetrical, and consistent block achieved with intrathecal local anesthesia, followed by gradual onset sympathectomy provided by epidural local anesthesia [88].

On placing the neuraxial block, it is advisable to decrease crystalloid fluid loading among patients with an elevated risk of pulmonary edema. It could also be helpful to start titrating a prophylactic vasopressor for maintaining the BP and SVR at maternal baseline, ensuring adequate coronary perfusion despite the neuraxial method utilized for anesthesia [111-113]. For instance, an infusion of phenylephrine and norepinephrine (starting titration 0.5-0.75 and 0.05-0.075 µg/kg/min, respectively) could be introduced by a peripheral IV line upon neuraxial block completion. Infusion should be titrated to maintain the heart rate above 60 bpm and the mean arterial pressure close to the baseline [114,115].

Intraoperative anesthesia monitoring techniques

The goals for managing intraoperative anesthesia include ensuring coronary perfusion, preventing tachycardia as well as ventricular end-diastolic excessive volume, maintaining myocardial contractility and CO, ensuring suitable levels of arterial oxygen, and preserving body temperature [83].

Effective hemodynamic monitoring is crucial for minimizing perioperative morbidity as well as mortality among cardiac parturients. Transesophageal echocardiography is a valuable tool for dynamically monitoring heart function among individuals with acute comorbidities or in situations where hemodynamic volatility is anticipated or arises during surgery. Among patients with myocardial ischemia without ECG changes, abnormalities in regional wall motion are frequently observed. Since mechanical anomalies in systole and diastole occur before electrical anomalies during ischemia, transesophageal echocardiography offers the benefit of early detection of cardiac ischemia [116]. The Vigileo/FloTrac system (Edwards Lifesciences, Irvine, CA, USA) offers valuable information regarding the hemodynamic status of the patient, including cardiac index, CO, stroke volume, and variation in stroke volume [117]. It offers an effective technique to assess the differential identification regarding circulatory failure, particularly to differentiate among vascular and cardiac factors, as well as blood volume. As a result, it offers a reliable technique for monitoring hemodynamic status, tracking clinical course changes, and evaluating the responses to treatment interventions among individuals with arterial catheters. This method may particularly be helpful and appropriate for guiding therapy with fluids and vasoactive medications in high-risk pregnancies [83].

Postoperative analgesia

The postnatal period poses a heightened risk for maternal morbidity and mortality related to CVD [83]. Postoperative pain is an important risk determinant regarding the development of MI and myocardial ischemia after surgery [118], and C-section can lead to moderate to acute pain after surgery. Inadequate pain control after surgery could delay the mother’s recovery, limit breastfeeding, and have an adverse impact on the mother-infant attachment [119]. Hence, for anesthesiologists, it is important to provide the best postoperative analgesia for pregnant women suffering from heart disease. Presently, multimodal analgesic approaches, such as peripheral nerve blockade, neuraxial anesthesia, and nonopioid analgesia, are the most common therapies employed following C-sections. Previous reports noted that the neuraxial anesthesia for females with AMI undergoing C-section, after discontinuation of antiplatelet drugs before surgery, was effective and safe postoperative analgesia and maintained more stable hemodynamic parameters [117,120]. For pregnant women receiving GA, postoperative patient-controlled analgesia with intravenous opioids combined with transversus abdominis plane block is an alternative to neuraxial analgesia. The ultrasound-guided transversus abdominis block offers successful analgesia for women undergoing C-sections, reduces the severity of nausea as well as vomiting, and ensures maternal satisfaction [121].

Postpartum period

The immediate postnatal period is characterized by a 60% increase in CO and a significant fluid shift due to autotransfusion from uterine contractions. For those patients suffering from AMI, it is an important transition period that requires close cardiovascular and hemodynamic monitoring in the coronary care unit. The third stage of labor involves a gradual infusion of prostaglandin and oxytocin analog (misoprostol) to prevent hypotension associated with the rapid infusion of oxytocin. Methylergonovine is related to HTN and vasoconstriction, making it contraindicated among patients with AMI [7].

The decision to breastfeed must be tailored for all patients. Growing evidence indicates that breastfeeding offers short- and long-term advantages for mothers regarding their cardiovascular health, including a decrease in the risk of developing HTN [122,123]. Although the decision regarding breastfeeding must be individualized, considering factors such as the etiology of AMI, the requirement for medication that may be contraindicated during breastfeeding, and the patient’s cardiovascular health must be taken into account [124,125].

The shift to ongoing postnatal care in an outpatient setting must involve coordination between perinatologists, obstetricians, and cardiologists, with an emphasis on supporting maternal recovery, medication management, addressing comorbidities, and assisting the patient in balancing newborn care during her recovery from an AMI associated with pregnancy [7].

The postnatal as well as post-MI periods are considered independent risk factors for the development of depression, with about 10% of women experiencing postnatal depression [126], while 18% of women develop depression following an MI [127]. Moreover, depression is linked to inadequate adherence to post-MI therapy recommendations [128]. Therefore, early postnatal evaluation, identification, and treatment of depression among females with pregnancy-related acute MI is crucial [7].

Cardiac rehabilitation must be advised for all patients following an AMI, as it provides substantial mental, as well as physical, health benefits. Outpatient cardiology visits provide an opportunity to adjust cardiac medications based on the etiology, signs, and ventricular function of the patient. Counselling regarding preventable risks, such as tobacco cessation, is important. The consensus statements regarding SCAD highlight that all individuals diagnosed with SCAD should be evaluated for other arterial anomalies, such as aneurysms, dissections, or fibromuscular dysplasia. Additionally, patients with SCAD should be counselled regarding recurrence risk, which ranges from 10% to 29% [129]. While one study demonstrated an insignificant recurrence rate among individuals on beta-blockers and a higher recurrence rate among those with HTN [130], SCAD recurrence, as well as prevention, approaches continue to be an area requiring further research [131].

The obstetrician or maternal-fetal medicine staff should initiate discussions on family planning and contraception, providing information regarding sterilization as well as long-acting contraception options. Generally, estrogen-containing contraception is not advised for patients following an AMI due to its increased thrombosis risk. However, WHO and US Medical Eligibility Criteria advise caution when considering the progesterone-containing implant technique for women with IHD [132]. Evidence suggests that the etonogestrel implant and levonorgestrel-releasing intrauterine methods are safe for women with CVD and are being utilized more frequently among these patients [133]. A shared decision-making process with these patients should focus on deciding on a technique that is secure, highly effective, and consistent with their goals as well as values [7].

Maternal and neonatal outcomes

Although maternal death due to acute MI has improved during recent decades, it is important to remember that women still face a higher risk of experiencing other complications [14]. Ventricular tachycardia, heart failure, and cardiac arrest are the most frequent subsequent heart problems reported for women experiencing AMI during pregnancy. One study found that more than a quarter of women with acute MI required admission to the ICU [134]. Among women diagnosed with SCAD, cardiogenic shock occurs in approximately one-quarter of them, with an equal proportion requiring mechanical support devices [26].

Perinatal outcomes are closely associated with maternal outcomes; however, the overall rate of perinatal mortality among women with acute MI is reported to be 4%. The majority of these cases can be linked to poor maternal outcomes; however, several studies indicate an elevated rate of prematurity that is primarily iatrogenic [135]. It is at least three times greater than the baseline prematurity rate for healthy women [136]. There appears to be no strong association between IHD and infants born small for gestational age (<10%), as opposed to what has been observed in women who have other types of coronary disease [137].

The pregnancy after an AMI may be associated with increased maternal morbidity as opposed to significant mortality [87]. Generally, future morbidity is assessed through infarction size and cardiac function before surgery. Women should be reassessed before proceeding with a future pregnancy to evaluate for progressive heart disease or to identify if left ventricular function has been impaired by the initial infarct. It has been suggested that women should be evaluated by echocardiography, as well as exercise testing, before a future pregnancy for the assessment of residual ischemia and left ventricular function [138]. Exercise testing may yield an increased rate of false-positive results requiring additional investigations. Burchill et al. demonstrated a two percent risk for heart failure among women with a history of IHD, and they found one maternal mortality during their study due to cardiac arrest [135].

Future pregnancy

Among women with a prior history of PAMI who plan future pregnancies, a complete cardiovascular examination is necessary to minimize the risks of maternal as well as fetal complications [69]. The evaluation of maternal heart risk stratification should be carried out through a comprehensive review of medical history, physical examination, lab analysis, electrocardiography, stress testing, echocardiography, and estimation of functional capacity [139,140].

Individuals with a prior history of IHD have an elevated risk of developing complications. Several studies have reported that an almost 10% incidence of ACS event recurrence is experienced by females with IHD during pregnancy [31]. However, this risk differs depending upon the underlying etiology of atherosclerotic disease, which results in an enhanced risk for cardiovascular complications, and a maximum recurrence rate of 20% is found with a prior history of spontaneous coronary dissection. The obstetric complication rate could be as high as 58%, which includes an elevated rate for C-sections, maternal HTN, premature delivery, and low birthweight [23]. Overall risk for maternal mortality is found to range from 0%-23% among various studies [141]. Additional significant predictive factors described during the Cardiac Disease in Pregnancy Study (CARPREG-II) trial comprise previous cardiac episodes, arrhythmias, a baseline New York Heart Association category III-IV heart failure or cyanosis score, valvular disease, ventricular malfunction, arthropathies, and pulmonary HTN [140].

The decision for a future pregnancy must be based upon a collaborative decision-making process in which multidisciplinary staff, patients and their families, maternal-fetal specialists, obstetricians, clinical pharmacists, cardiologists, and primary care physicians should be included. Also, the discussion should include PAMI effects on the heart's present and future function and the anticipated outcomes, as well as the prognosis for both mothers and their fetuses. Women suffering from residual IHD or SCAD must be strongly advised to avoid future pregnancies [142].

The cardiovascular risk determinants, for example, DM, HTN, diet, and tobacco usage, should be improved before conception. The medication must also be revised and managed as its compatibility relates to continuing the pregnancy. Delays regarding cardiovascular evaluation are related to further adverse cardiac outcomes [140]. Several experts have recommended CA for individuals at elevated risk for the assessment of heart disease severity and development [143]. Although studies found no difference in the incidence and outcomes of ischemic episodes during pregnancies among females with a prior history of IHD who experience pre-pregnancy revascularization when compared to women with no pre-pregnancy reperfusion [23].

Case studies and outcomes

A study done by Uysal et al. [144] reported that a woman 38 years of age at 37 weeks of gestation was admitted to the hospital with chest pain. Previously, she had undergone mitral valve replacement. Her clinical presentation and ECG findings were consistent with STEMI, and the thrombus was observed to be on a posterolateral segment of the circumflex artery through angiography. This patient was managed with medical therapy and had a normal delivery with no complications. After follow-up, the mother and infants were discharged from care. The study concluded that emergency intervention must be taken for pregnant women with MI, which may be lethal for the mother and the infant.

A woman, 33 years of age at 31 weeks of gestation with twins, arrived at the health facility with recurrent chest tightness for the past 12 weeks, which had been worsening for the last week, and experiencing chest pain for the last four hours. The hypersensitive troponin and ECG results indicated acute STEMI. Although the woman had no associated clinical history, she had numerous risk factors, including age >30 years, hyperlipidemia, and assisted reproduction. Following diagnosis, the woman received antiplatelet and anticoagulant therapy. The C-section and CA carried out seven days later identified stenosis and thrombus in the RCA. After receiving medication, the woman’s health improved [145].

A woman who was 30 years of age presented at the health facility with abnormal ECG findings, which were identified during her routine medical check-up in the fifth week of twin pregnancy. She had no significant indications or previous history of cardiac disease. The woman has stable vital signs without other noticeable anomalies during her complete physical evaluation. The older siblings of the woman had a hypertrophic obstructive cardiomyopathy history, and in the past, she had experienced percutaneous radiofrequency ablation of the intramyocardial septum. Although the echocardiography findings of the patient did not indicate any evident anomalies. Subsequently, cardiac MRI on follow-up two years later confirmed hypertrophic cardiomyopathy [146].

Verhaert et al. described a case of a 27-year-old primigravida with an unremarkable clinical history and without CAD risk factors. At 28 weeks of gestation, she was treated for preterm labor with tocolytic therapy, starting with ritodrine and later transitioning to nifedipine. She also experienced non-STEMI. Her CA, which was carried out two days following her acute event, was normal. Her subsequent course was uneventful, resulting in a spontaneous vaginal birth at 40 weeks of gestation [147].

Ryshten et al. presented a case report of a woman who was 37 years old, without any cardiovascular risk factors, who survived an AMI due to multivessel SCAD due to cardiogenic shock during the third postpartum week [148]. With medical treatment along with angioplasty, she recovered completely.

Jaiswal and colleagues described an optimum management plan by describing the management of a 45-year-old woman with STEMI [149]. They suggested early utilization of CA for the identification of major pathology among such patients, with a preference for radial artery access. Pregnant women with AMI due to atherosclerotic disease should receive aspirin, 325 mg, clopidogrel, 600 mg, and either BMS or balloon angioplasty should be carried out for revascularization. PCI with heparin should be preferred to bivalirudin and reserved for those patients who experience acute heparin allergy.

Makkonen et al. [150] described a case of a 38-year-old expectant woman for whom the risk determinants for CAD included a history of preeclampsia and cigarette smoking during a prior pregnancy, and an acute anterior MI that occurred during the 18th week of the reported pregnancy. During the 28th week of the pregnancy, CA demonstrated critical stenosis within the coronary arteries. She experienced normal labor without complications at term.

A study was undertaken by Vinatier et al. to assess the risks and prognosis for pregnancy among women who experienced an MI and to suggest guidelines regarding pre-pregnancy counseling as well as clinical supervision for pregnancy and delivery [151]. The study revealed that the majority of pregnancies developed satisfactorily if cardiovascular issues were identified and treated quickly. During the pregnancy, adequate rest was required, while activities or situations that could increase the myocardial workload should be avoided. Epidural anesthesia was one of the preferred techniques for normal vaginal birth in this cohort.

Gil et al. described a case of a 31-year-old expectant woman at 38 weeks of gestation. She had a family history of hyperlipidemia and cardiac disease [152]. She was suffering from a septal AMI. The C-section was conducted under general anesthesia. The patient was subsequently transferred to the ICU. There were no complications during or after surgery. Echocardiography demonstrated septal hypokinesia with normal systolic function, while CA demonstrated normal coronary vessels. The patient remained hemodynamically stable and was discharged from the ICU after 48 hours.

Duarte et al. described a case of a female patient at 31 weeks of gestation. She had a prior history of alcoholism, smoking, and HTN and was admitted to the hospital after an episode of syncope [120]. At the time of admission, she was found to be hypotensive. Her ECG demonstrated marked ST-elevation in leads 1, AVL, and V1-V6. Increased cardiac enzyme levels were observed. TTE demonstrated a decrease in the contractility of the septum as well as the left ventricle, and the LVEF was 30%. Angiography highlighted a proximal obstruction in the LAD artery. A BMS was inserted after balloon angioplasty, but was unsuccessful. Treatment was initiated with aspirin, clopidogrel, and beta-blockers. It was decided to perform a C-section four weeks following the MI. Seven days before delivery, clopidogrel was discontinued. Levosimendan was started one day before delivery with improvement of her heart function. The C-section was carried out utilizing an epidural block. There were no intraoperative complications other than mild hypotension, which was managed easily by administering phenylephrine.

Balmain and associates reported a case of a 40-year-old expectant female patient who was admitted with an AMI complicated by ventricular fibrillation [153]. The patient successfully underwent primary PCI. As maternal age is higher in the well-developed nations, AMI during pregnancy could become more common.

A woman at 32 weeks of gestation arrived at a pre-hospital setting with severe chest pain due to an RCA dissection. Pre-hospital analysis of AMI was conducted by the ambulance staff utilizing a 12-lead ECG. She was successfully treated with PCI and stenting [154].

Boztosun et al. presented the case of an AMI in a woman 43 years of age at 20 weeks of gestation, who was effectively treated with clopidogrel, aspirin, and intracoronary stenting without complications [155]. A study was performed by Tweet et al. to evaluate the presentation, significant factors, and results for pregnancy-associated SCAD (P-SCAD) when compared with SCAD not associated with pregnancy (NP-SCAD) [26]. The study highlighted that most of the events took place during the first month after delivery (35/50, 70%). When compared with the NP-SCAD, those with P-SCAD more commonly presented with the STEMI (57% versus 36%, respectively), left main/multivessel SCAD (24% versus 5% and 33% versus 14%, respectively), and the left ventricular function as high as 35% (26% versus 10%). In women who underwent imaging for other vascular areas, the occurrence of P-SCAD was found to be less frequent among those identified with fibromuscular dysplasia or abnormalities in the extracoronary blood vessels (42% versus 64%, and 46% versus 77%, respectively). The women with P-SCAD were mostly multiparous, had a previous history of preeclampsia, and had received infertility treatment. On the long-term follow-up (mean 2.3 years), repeated SCAD was noticed among 51 individuals, while there was no difference for five-year recurrence rates on Kaplan-Meier analysis (10% versus 23%). The study concluded that P-SCAD women had more severe presentations as well as high-risk characteristics than females with NP-SCAD.

Wang et al. reported a case of a woman aged 26 years with STEMI at 14 weeks of gestation [156]. CA demonstrated no abnormalities. The patient had no history of coronary risk factors, including DM, smoking, HTN, and dyslipidemia. However, direct evidence of coronary spasm was observed in this woman, and numerous factors suggested that the coronary spasm was an important cause of her MI. It was also suspected that hyperthyroidism played a significant part in her coronary spasm. Timely utilization of CA can help identify lesion types in the coronary artery.

Moussa et al. presented a case of pregnancy-associated AMI due to SCAD in a 37-year-old woman who arrived at an emergency department with a complaint of dyspnea and substernal chest pain with an abrupt onset that occurred eight days following the birth of premature twins [157]. Cardiac catheterization demonstrated 75%-90% narrowing of the LMCA, expanding in the proximal as well as mid portions of the LAD artery. A left heart catheterization done in an emergency setting revealed LMCA vasospasm, which did not respond to medical therapy. A second coronary artery catheterization found a dissection necessitating critical coronary artery bypass grafting involving three vessels: the circumflex, LAD, and LMCA. She subsequently had an uneventful recovery.

A 38-year-old woman, who was a great-grand multipara, arrived at the emergency department with sudden-onset chest pain. She had a longstanding history of HTN and was a habitual smoker. It was discovered incidentally that she was five weeks pregnant and diagnosed with AMI, which was treated with primary PCI. Subsequently, her pregnancy was complicated by chronic HTN that was poorly managed, but at 36 weeks of gestation, she delivered a healthy baby successfully [158].

Hayashi et al. reported a primigravida female with AMI due to CAS induced by methylergometrine maleate administered intravenously just following delivery. Despite the frequent utilization of ergot derivatives to stimulate uterine contractions, cardiac problems associated with this medicine are uncommon. Although the MI may be ignored among young women during the early postpartum period, timely assessment and careful monitoring must be carried out [159].

Arimura et al. reported a case of a 43-year-old, primiparous woman who had an AMI. She successfully underwent primary PCI. Elective C-section was uneventfully carried out at 32 weeks of gestation [160].

De Santis et al. described a case of a 36-year-old woman at nine weeks of gestation with a history of persistent HTN, CAD, and dyslipidemia who required antiplatelet therapy. Aspirin and clopidogrel were given until one week before delivery, and she had a healthy infant at 36 weeks of pregnancy by C-section with no complications [161].

A Caucasian pregnant female patient aged 33 years arrived at the emergency room with a one-hour history of abrupt onset, acute angina that started when she was shopping. The pain radiated to her back, arm, and left shoulder. Also, she complained of dyspnea, dizziness, vomiting, nausea, and sweating. An anterolateral MI diagnosis was performed, and she was administered aspirin, clopidogrel, and I/v heparin while the necessary arrangements were carried out for an urgent interfacility transfer to perform the primary PCI. The cardiac angiography demonstrated atheroma obstructing the proximal portion of the LAD artery, while the circumflex and RCA arteries appeared normal. Angioplasty and BMS were carried out for the LAD lesion. The woman was later shifted from the ICU to the maternity unit, where she completely recovered and successfully underwent an elective C-section at 39 weeks of gestation [162].

Shahabi et al. reported two cases of SCAD, one at 34 weeks of gestation and the other after delivery [163]. Both women were identified with angiography and treated medically. One patient required PCI and recovered cardiac function with an optimum obstetric outcome.

Lameijer et al. reviewed the CA records from a Dutch teaching health facility to identify women who arrived with AMI either during pregnancy or after delivery [164]. Two patients were found. Both women were older than 30 years of age and experienced MI during the postpartum period. One woman’s AMI occurred due to coronary artery blockage, and the other was due to coronary artery dissection. Both women were treated with PCI and survived.

Gieres et al. reported a case of a 30-year-old primipara who had no significant previous medical record [165]. She was admitted to the hospital for labor following an intrauterine fetal demise after 37 weeks of pregnancy. After medically induced labor, the woman experienced convulsive fits and suffered cardiopulmonary arrest. Temporary mechanical cardiopulmonary resuscitation was carried out, and impulsive circulation was restored. A diagnosis of amniotic fluid embolism was made based on the concurrent occurrence of severe vaginal bleeding and STEMI. Lab findings met the criteria regarding DIC, prompting mechanical hemostasis as well as hemostatic resuscitation. The transesophageal cardiac ultrasound highlighted hypokinesia to inferior wall akinesia. CA was not possible due to persistent DIC. After transfer of the patient to the ICU, ST-segment elevation was noted, and the MI was managed medically. A cardiac MRI performed three months after the event showed myocardial fibrosis in two different regions. Based on the cardiac artery structure observed during the chest CT scan, the infarcted regions corresponded to two distinct coronary supply regions.

Zieliński et al. reported on a 23-year-old woman at 32 weeks of gestation, treated for hypothyroidism and insulin-dependent DM, who was brought to the hospital due to severe chest pain [166]. An ECG showed ST-segment elevation in leads I, AVL, and V4-V6, and increased troponin-T levels. She was diagnosed with an MI, initiated on a low-molecular-weight heparin infusion, and then had primary coronary angioplasty along with revascularization performed. Following the procedure, she received DAPT with clopidogrel and acetylsalicylic acid. Her pregnancy was ended at 38 weeks of gestation with an elective C-section and delivery of a healthy baby.

## Conclusions

During pregnancy, MI is an uncommon occurrence; however, CVD remains a significant cause for maternal as well as fetal mortality. Women with comorbidities and risk factors should undergo a thorough assessment to better predict the pregnancy outcome. For women with comorbidities and risk factors who become pregnant, early diagnosis is crucial for predicting outcomes. A multidisciplinary team approach involving obstetricians, anesthesiologists, and cardiologists is essential for developing a management plan, and appropriate care will play a key role in determining future prognosis.
